# Relationship between patient-perceived quality of primary care and self-reported hospital utilisation in China: A cross-sectional study

**DOI:** 10.1080/13814788.2024.2308740

**Published:** 2024-02-26

**Authors:** Chenwen Zhong, Junjie Huang, Lina Li, Zhuojun Luo, Cuiying Liang, Mengping Zhou, Nan Hu, Li Kuang

**Affiliations:** a Department of Health Policy and Management, School of Public Health, Sun Yat-sen University, Guangzhou, Guangdong, China; b Jockey Club School of Public Health and Primary Care, The Chinese University of Hong Kong, Hong Kong, China; c Department of Medical Epidemiology and Biostatistics, Karolinska Institutet, Solna, Sweden; d Department of Family and Preventive Medicine, and Population Health Sciences, University of UT School of Medicine, Salt Lake City, UT, USA

**Keywords:** Primary care, quality, assessment survey of primary care, self-reported hospital utilisation, hospital admission, outpatient visits, emergency department visits

## Abstract

**Background:**

Reducing avoidable hospital admissions is a global healthcare priority, with optimal primary care recognised as pivotal for achieving this objective. However, in developing systems like China, where primary care is evolving without compulsory gatekeeping, the relationship between patient-perceived primary care quality and hospital utilisation remains underexplored.

**Objectives:**

This study aimed to explore the association between patient-perceived primary care quality and self-reported hospital utilisation in China.

**Methods:**

Data were collected from 16 primary care settings. Patient-perceived quality of primary care was measured using the Assessment Survey of Primary Care scale across six domains (first-contact care, continuity, comprehensiveness, accessibility, coordination, and patient-centredness). Hospital utilisation included patient self-reported outpatient visits, hospital admissions, and emergency department (ED) visits in the last six months. Logistic regression analyses were examined associations between self-reported hospital utilisation and perceived primary care quality adjusted for potential confounders.

**Results:**

Of 1,185 patients recruited, 398 (33.6%) reported hospital utilisation. Logistic regression analyses showed that higher total scores for patient-perceived quality of primary care were associated with decreased odds of hospital utilisation (adjusted odds ratio(AOR): 0.417, 95% confidence interval (CI): 0.308–0.565), outpatient visits (AOR: 0.394, 95% CI: 0.275–0.566) and hospital admissions (AOR: 0.496, 95% CI: 0.276–0.891). However, continuity of care was positively associated with ED visits (AOR: 2.252, 95% CI: 1.051–4.825).

**Conclusion:**

Enhanced patient-perceived quality of primary care in China is associated with a reduction in self-reported overall hospital utilisation, including outpatient visits and hospital admissions. However, better continuity of care may be associated with increased ED visits. Further research is warranted for precise insights and validation of these findings.

## KEY MESSAGES

Improved patient-perceived primary care quality in China was linked to lower self-reported hospital utilisation, including outpatient visits and hospital admissions.

Better primary care accessibility was associated with decreased self-reported emergency department visits and outpatient visits.

Continuity of care showed a positive association with emergency department visits, warranting further research.

## Introduction

Primary care is the cornerstone of the community, providing accessible and comprehensive health services with strong coordination and a continuous doctor--patient relationship throughout different episodes of illness [1]. Globally, robust primary care systems correlate with better health outcomes, lower mortality rates, and increased satisfaction [2]. Addressing unnecessary hospital utilisation is a global priority [3]. In the UK, avoidable emergency admissions for chronic conditions cost the National Health Service £1.42 billion [4]. Enhanced primary and community services were estimated to curtail these costs by 8–18% [4]. In the US, increased availability of primary care correlated with better health outcomes and reduced costly healthcare utilisation like hospitalisations and ED visits [5].

Researchers have been exploring mechanisms underpinning the link between primary care and hospital utilisation, especially in the core primary care domains, including first-contact care, continuity, comprehensiveness, accessibility, coordination, and patient-centred care [6]. A systematic review revealed that continuity of care in countries like the UK, the US, Canada, and Europe correlates with fewer ED visits and admissions [7]. Increased accessibility to primary care services was also found to have potentially reduced avoidable admissions [8]. However, the applicability of these findings to less developed primary care systems, such as China, remains uncertain.

China’s primary care system comprises diverse facilities, including community health centres (CHCs), community health stations, township hospitals, village clinics, and outpatient departments/clinics [9]. These facilities are crucial in providing essential services like health promotion, disease prevention, diagnosis, chronic disease management, maternal and child health care, and essential emergency services [10]. By 2019, approximately 90% of Chinese households had access to primary care facilities within a 15-minute distance [11]. In 2022, the government issued the ‘Opinions on Promoting the High-quality Development of Family Doctor Contract Services’ to broaden the reach of family doctor contracted services to gradually establish a system where family doctors serve as the gatekeepers of health [12]. In specific cities like Shenzhen and Dongguan, a non-compulsory gatekeeper system is operational and guarded by insurance reimbursement. As part of efforts to encourage patients to utilise primary care facilities, health insurance covers all or a significant portion of the charges when patients seek care from a primary care facility or are referred by the designated primary care provider. Alternatively, patients bear the charges out-of-pocket [13]. Despite these efforts, the current absence of a compulsory gatekeeping system allows patients to choose medical practitioners and institutions directly, raising concerns about overutilisation and unnecessary ED visits even for minor ailments [14].

Our prior population-based studies in Guangdong Province and nationwide emphasised the crucial role of primary care intensity in health outcomes such as maternal and child health and medical expenditures [15, 16]. These studies highlighted primary care’s contribution to healthcare, advocating for policy actions to enhance primary care nationwide. Using individual-level data from multiple waves of the China Health and Retirement Longitudinal Study (CHARLS) [17], we found that individuals with primary care consultations had significantly lower odds of ED visits, hospitalisation, and shorter hospitalisation days. Nevertheless, the underlying mechanisms driving the connection between primary care and hospital utilisation remain uncharted.

In this context, our study aims to investigate the association between patient-perceived primary care quality and self-reported hospital utilisation in China, focusing on outpatient visits, admissions, and ED visits within China’s healthcare system. Building on prior experiences and literature, we hypothesise a negative association. Specifically, we anticipate that improved primary care performance across domains is likely to be linked with reduced instances of patient self-reported hospital utilisation.

## Methods

### Study design and sample size calculation

This cross-sectional study was conducted between January and March 2019 in ten cities in Guangdong province, China. Based on prevalence rates from a prior study [17] indicating 45% for outpatient visits, 27% for admissions, and 4% for ED visits, a sample size of 1,027 was determined to achieve a significance level of 0.05 and a power of 0.80 [18]. Accounting for potential non-responses, 15% more participants were included, resulting in a total sample size of approximately 1,181 individuals.

### Participant recruitment

We employed a multi-phase sampling approach to identify potentially eligible patients from 12 medical institutions, covering 16 primary care settings in Guangdong Province. Verbal informed consent was acquired from all patients for specified reasons. Ethical approval was granted by the Institutional Review Board of the School of Public Health, Sun Yat-Sen University, Guangzhou, China (Approval No: 2018.014). Further details are available in Appendix 1.

### Data collection

Data collection was conducted in the waiting areas of the primary care settings. Eligible participants were approached by our investigators directly and were interviewed after their consultations, upon their agreement to participate. A team of five investigators conducted all interviews, each holding a Bachelor’s degree in Medicine. These investigators are postgraduate students with a robust background in primary care, and they underwent advanced training before embarking on the project. After the interview, each questionnaire was reviewed to ensure completeness. Questionnaire completion typically took 20–25 min.

### Quality assurance

We employed face-to-face one-on-one interviews between investigators and patients to ensure questionnaire response quality. This method aimed to enhance diligence, minimise omissions, and prevent response conformity. Post-questionnaire completion, investigators conducted immediate checks to confirm accurate and complete entries. Quality control measures also included intensive investigator training, standardising on-site procedures and maintaining consistency in interpreting and presenting questionnaire items. Data entry accuracy was verified through a 15% random check.

The questionnaire featured four sections: (i) sociodemographic characteristics, (ii) patient health status, (iii) patient previous primary care utilisation and (iv) items in the Assessment Survey of Primary Care (ASPC) scale. Socio-demographic data included gender, age, marital status, family monthly household income, employment status, household status and health insurance. Health status questions assessed chronic disease diagnosis, the number of chronic diseases (if any), and self-rated health. Primary care utilisation details included years since the first visit, the number of visits in the last two weeks, and whether having a family doctor.

### ASPC scale

This study used the ASPC scale to assess patients’ perceptions of primary care quality [19]. Prior validation, documented in a peer-reviewed journal, confirmed the scale’s reliability and validity within our dataset [19]. Aligned with global primary care standards, the ASPC scale evaluates first-contact care, accessibility, continuity, comprehensiveness, coordination and patient-centred care across 41 items [19]. Respondents, rated items on a four-point Likert scale (1 = never, 2 = sometimes, 3 = often and 4 = always). A neutral ‘not sure’ option (scored 2.5) was available for questions with limited knowledge. The score for each domain was calculated by averaging embedded item scores, while the total score reflecting patient-perceived quality of primary care emerged from averaging all six-domain scores. Further scale details are shown in Appendix 2.

### Patient self-reported hospital utilisation

Patients self-reported their hospital utilisation, including outpatient visits, admissions and ED visits, over the last six months. This time frame was chosen to balance the need for recent data with participants’ capacity to recall their experiences accurately, ensuring the reliability of the gathered information. A positive response was recorded as ‘yes’ if a patient reported any encounter with outpatient, admission, or ED services in the last six months. To provide a comprehensive binary representation of all hospital-based healthcare interactions, self-reported overall hospital utilisation was recorded as ‘yes’ if any form of hospital utilisation occurred during this period.

### Data analysis

The ASPC scale’s total score and domain scores were presented as mean ± standard deviation (SD) while categorical variables were expressed as frequency with percentage. Pearson chi-square tests were used to analyse bivariate associations between patient characteristics and self-reported overall hospital utilisation. Independent t-tests identified differences in patient-perceived quality of primary care between those with or without self-reported overall hospital utilisation. Logistic regression analyses were employed to investigate the association between patient-perceived quality of primary care and patient-self-reported hospital utilisation. Patient utilisation was the dependent variable, with ASPC scale domain scores and total score serving as independent variables. Adjustments were made for patient socio-demographic characteristics, health status and primary care utilisation. Results were presented as adjusted odds ratios (AORs) with corresponding 95% confidence intervals (CIs) and *p*-values. These statistics were used to quantify the likelihood of patient self-reported hospital utilisation associated with changes in the ASPC total score or each domain. Statistical significance was determined at *p* < 0.05. Statistical analyses employed SPSS 26.

## Results

### Population characteristics

This study included 1,185 patients, detailed in Table 1. Of these, 398 (33.6%) reported having any type of hospital utilisation in the last six months. Outpatient visits, admissions and ED visits were reported by 287, 78 and 19 patients, respectively. Additionally, 15 patients reported hospital utilisation for other reasons such as prescription renewals or routine check-ups. Detailed hospital utilisation and the number of times for each instance are shown in Table 2. Concerning the possible differences between cities, while patient characteristics varied across the ten cities, there were no significant differences in self-reported overall hospital utilisation. Additional details are in Appendices 3 and 4.

**Table 1.** Patient socio-demographic characteristics, health status and primary care utilisation.

	Hospital utilisation during the past six months		*p* ^#^
	Yes (*N* = 398)	No (*N* = 787)	
Gender			0.185
Male	141(35.4)	310(39.4)	
Female	257(64.6)	477(60.6)	
Age(years)			0.685
18–30	69(17.3)	118(15.0)	
31–50	139(34.9)	270(34.3)	
51–70	125(31.4)	267(33.9)	
>70	65(16.3)	132(16.8)	
Marital status			0.662
Unmarried	29(7.3)	53(6.6)	
Married, divorced or widowed	369(92.7)	735(93.4)	
Education			0.005**
Primary school or below	90(22.6)	224(28.5)	
Middle/high school	213(53.5)	432(54.9)	
Bachelor’s degree or above	95(23.9)	131(16.6)	
Employment status			0.437
Employed	188(47.2)	390(49.6)	
Retired	96(24.1)	199(25.3)	
Unemployed	114(28.6)	198(25.2)	
Monthly household Income (CNY)			0.099
≤5,000	285(71.6)	603(76.6)	
5001–10,000	62(15.6)	112(14.2)	
>10,000	51(12.8)	72(9.1)	
Household status			0.216
Local residence	239(60.1)	443(56.3)	
Non-local residence	159(39.9)	344(43.7)	
Health insurance			0.471
Yes	358(89.9)	697(88.6)	
No	40(10.1)	90(11.4)	
Health status			<0.001***
Poor	75(18.8)	82(10.4)	
General	195(49.0)	344(43.7)	
Good	128(32.2)	361(45.9)	
Number of chronic diseases			0.002**
0	190(47.7)	459(58.3)	
1	132(33.2)	217(27.6)	
2 and above	76(19.1)	111(14.1)	
Number of years since first visit to the primary care setting			0.802
< 1	39(9.8)	80(10.2)	
1–5	153(38.4)	287(36.5)	
> 5	206(51.8)	420(53.4)	
Number of visits to the primary care setting in the past two weeks			0.002**
0	44(11.1)	129(16.4)	
1	241(60.6)	498(63.3)	
2 and above	113(28.4)	160(20.3)	
Whether have a family doctor			0.533
Yes	152(38.2)	286(36.3)	
No	246(61.8)	501(63.7)	

*Values are numbers (percentages); ^#^*p* values associated with Pearson’s chi-square tests.

**Table 2.** Patient self-reported hospital utilisation in the last six months.

	Self-reported outpatient visits	Self-reported hospital admissions	Self-reported emergency department visits	Self-reported overall hospital utilisation
No	898(75.8)	1,107(93.4)	1,166(98.4)	787(66.4)
Yes	287(24.2)	78(6.6)	19(1.6)	398*(33.6)
Only 1 visit	159(55.4)	64 (82.1)	16(84.2)	–
2–5 visits	107(37.3)	12(15.4)	3(15.8)	–
Over 6 visits	21(7.3)	2(2.6)	0(0.0)	–

*Values are numbers (percentages). –: Not applicable as the overall hospital utilisation was expressed as a dichotomous variable. When patients had encountered any type of hospital utilisation in the last six months, the overall hospital utilisation was considered as ‘yes’, otherwise ‘no’. *In addition to outpatient visits, hospital admissions, and emergency department visits, the remaining 14 patients reported hospital utilisation for reasons such as prescription renewals or routine health check-ups. They were not analysed separately in our study due to limited data.

### Patient-perceived quality of primary care

The differences in patient-perceived quality of primary care between those with or without hospital utilisation in the last six months are shown in Table 3. The scores for patient-perceived quality of primary care across different cities are presented in Appendix 5. The total ASPC score averaged at 2.79 ± 0.44, while individual domain scores ranged from 2.38 ± 0.66 to 3.17 ± 0.66. Patients who reported no overall hospital utilisation in the past six months showed statistically higher total scores and scores in the domains of comprehensiveness, coordination and patient-centred care compared to those reporting specific hospital utilisation during the same period (*p* < 0.05).

**Table 3.** Patient-perceived quality of primary care using the Assessment Survey of primary care scale and the difference between patients with or without self-reported overall hospital utilisation during the past six months.

Domains	All participants^	Self-reported overall hospital utilisation during the past six months		Difference^&^	*p* ^#^
		Yes^ (*N* = 398)	No^ (*N* = 787)		
First-contact care	3.06 ± 0.64	3.02 ± 0.69	3.08 ± 0.62	0.06 ± 0.04	0.123
Continuity	2.46 ± 0.69	2.44 ± 0.68	2.46 ± 0.7	0.02 ± 0.04	0.575
Accessibility	3.17 ± 0.66	3.12 ± 0.68	3.20 ± 0.65	0.09 ± 0.04	0.032*
Comprehensiveness	2.38 ± 0.66	2.28 ± 0.67	2.43 ± 0.65	0.16 ± 0.04	<0.001***
Coordination	2.71 ± 0.52	2.41 ± 0.62	2.85 ± 0.39	0.44 ± 0.03	<0.001***
Patient-centred care	2.99 ± 0.65	2.92 ± 0.67	3.03 ± 0.63	0.11 ± 0.04	0.005**
Total score	2.79 ± 0.44	2.70 ± 0.45	2.84 ± 0.42	0.15 ± 0.03	<0.001***

*Note*: ^Values are means ± standard deviations; &: Values in difference are means ± standard errors, reflecting the variation in patients’ perceived primary care quality. A difference value > 0 indicates higher patient-perceived quality of primary care in patients who reported no hospital utilisation in the past six months compared to those reporting specific hospital utilisation during the same period. #*p* values associated with independent sample t-tests. *: *p* < 0.05, **: *p* < 0.01, ***: *p* < 0.001.

Table 4 presents logistic regression results investigating the associations between patient-perceived quality of primary care and self-reported hospital utilisation in the last six months. An elevated patient-perceived primary care quality score was associated with decreased self-reported overall hospital utilisation (AOR: 0.417, 95% CI: 0.308–0.565), outpatient visits (AOR: 0.394, 95% CI: 0.275–0.566) and hospital admissions (AOR: 0.496, 95% CI: 0.276–0.891). Specific domains such as first-contact care, comprehensiveness, coordination and patient-centred care demonstrated negative associations with overall hospital utilisation.

**Table 4.** Relationships between primary care assessment score and hospital utilisation in the last six months using logistic regression analyses (*N* = 1,185).

ASPC scale	Self-reported overall hospital utilisation AOR(95%CI)	Self-reported outpatient visits AOR (95%CI)	Self-reported hospital admissions AOR (95%CI)	Self-reported emergency department visits AOR (95%CI)
Total score	0.417(0.308,0.565)***	0.394(0.275,0.566)***	0.496(0.276,0.891)*	0.750 (0.247,2.277)
First-contact care	0.811(0.666,0.989)*	0.874(0.691,1.105)	0.847(0.581,1.235)	1.133(0.528,2.431)
Continuity	0.917(0.761,1.105)	0.936(0.743,1.179)	0.956(0.658,1.389)	2.252(1.051,4.825)*
Accessibility	0.849(0.706,1.023)	0.838(0.665,1.058)	0.915(0.612,1.366)	0.333(0.169,0.656)**
Comprehensiveness	0.695(0.572,0.843)***	0.715(0.571,0.894)**	0.760 (0.523,1.106)	0.715(0.350,1.458)
Coordination	0.143(0.106,0.195)***	0.125(0.088,0.177)***	0.234(0.148,0.371)***	1.059(0.429,2.619)
Patient-centred care	0.729(0.597,0.890)**	0.702(0.556,0.886)**	0.830(0.564,1.222)	0.975(0.450,2.113)

*Note*: ASPC scale: Assessment Survey of Primary Care scale. *: *p* < 0.05, **: *p* < 0.01, ***: *p* < 0.001. AOR: adjusted odds ratio. Values highlighted in bold signify statistical significance.

1. Logistic regression analyses were performed to evaluate the relationship between total score and score of each and each type of hospital utilisation in the last six months.

2. Dependent variables were patient self-reported hospital utilisation (Yes/No). Independent variables were the total score or score of each domain in ASPC scale. Confounders were adjusted for patient gender, age, marital status, family monthly household income, employment status, household status, health insurance, cities of recruitment, number of chronic diseases, health status, the number of years since first visit to the primary care setting, the number of visits to the primary care setting, and whether had a family doctor.

3. An AOR < 1 indicated a negative association, implying that an increased score in ASPC scale (total score or score of each domain) is negatively associated with the likelihood of hospital utilisation in last six months.

Considering specific utilisation types, comprehensiveness, coordination and patient-centred care were linked to fewer outpatient visits. A higher coordination score correlated with fewer hospital admissions (AOR: 0.234, 95% CI: 0.148–0.371). More accessible primary care was associated with a decreased likelihood of ED visits (AOR: 0.333, 95% CI: 0.169–0.656). Continuity of care positively associated with ED visits (AOR: 2.252, 95% CI: 1.051–4.825).

## Discussion

### Main findings

This cross-sectional study explored the relationship between patient-perceived quality of primary care and self-reported hospital utilisation in the last six months. Results indicated that a higher total score for patient-perceived quality of primary care was associated with decreased self-reported overall patient hospital utilisation (AOR: 0.417, 95% CI: 0.308–0.565), particularly for outpatient visits (AOR: 0.394, 95% CI: 0.275–0.566) and hospital admissions (AOR: 0.496, 95% CI: 0.276–0.891). This association was consistent across various domains, including first-contact care, comprehensiveness, coordination and patient-centred care. While improved care coordination was associated with fewer hospital admissions, enhanced accessibility was linked to reduced ED visits. Nonetheless, continuity of care appeared to be associated with increased ED visits, which warrants closer examination.

### Comparison with existing literature

Our study aligns with prior research, indicating that robust primary care, as perceived by patients with higher scores, is associated with reduced high-cost hospital utilisation, specifically for outpatient visits and hospital admissions [20]. Enhancing first-contact care, comprehensiveness, coordination and patient-centred care is associated with reduced self-reported overall hospital utilisation. First-contact care can effectively modify patients’ health-seeking behaviour [21]. In this study, around 30% of Shenzhen and Dongguan participants, under non-compulsory gatekeeping guarded by insurance reimbursement, opted for primary care as their first point of contact due to substantial insurance reimbursement [13]. Higher comprehensiveness scores signify a comprehensive array of primary and public services addressing diverse patient needs [6], supported by a qualitative study in China highlighting patient appreciation of comprehensive primary care [22]. Furthermore, strong coordination ensures timely referrals and bolsters connections across healthcare system levels. This corresponds with a US qualitative study that recognises the essential role of care coordination in ensuring positive patient experiences [23]. Patient-centred care, a core principle of general practice, prioritises care responsive to patient preferences, needs and values, guiding clinical decisions [24]. These domains embody the fundamental principles of primary care, synergistically contributing to cumulative patient benefits and ultimately reducing hospital utilisation.

Logistic regression reveals that better coordination is linked to lower hospital admission risk, while enhanced accessibility to primary care corresponds to lower odds of ED visits. Effective coordination facilitates information exchange among providers through electronic health records, enabling timely interventions and treatment and potentially reducing disease progression and hospitalisations [25]. An international survey across 34 countries correlates accessible primary care with reduced ED visits [26]. Similar associations have been observed in the UK, where enhanced GP access lessened the burden of ED visits [27].

Unexpectedly, heightened continuity of care is associated with increased odds of ED visits, contrasting with previous research [28, 29]. This may be influenced by factors such as easy access to emergency services and acute exacerbations requiring immediate attention despite maintaining a robust continuity of primary care. Further empirical investigations are needed to understand this complex association fully.

### Implication of practice and research

Our study highlights the potential relationship between improving patient-perceived primary care and decreasing high-cost hospital utilisation, contributing to the global dialogue on evaluating such relationships, especially in countries embarking on primary care development. The implications of our findings extend to local practice and policy decisions, endorsing primary care utilisation and efficient gatekeeping to ease healthcare system burdens. Policymakers should sustain their prioritisation of primary care development in response to ageing populations and chronic diseases. Future research can affirm our findings through hospital audit data, providing a more comprehensive understanding of primary care and hospital utilisation dynamics.

### Strengths and limitations

This study has notable strengths, employing a robust ASPC scale encompassing six key domains to evaluate patient-perceived primary care quality comprehensively. The large sample size of 1,185 patients recruited from 12 medical institutions encompassing 16 primary care settings in China enhances the statistical power. The utilisation of logistic regression analyses, adjusting for various socio-demographic and health-related factors, strengthens the study’s internal validity. Furthermore, the focus on patient self-reported outcomes provides real-world insights into healthcare utilisation patterns.

Acknowledging limitations, the cross-sectional design precludes establishing causal relationships. Potential recall bias in self-reported hospital utilisation indicators suggests a need for future research to enhance validity by incorporating data from official records. While limited ED visits and a lack of detailed reasons for hospital utilisation are areas for improvement, they do not overshadow the study’s overall contributions. The absence of a defined minimal important difference (MID) in the ASPC scale is a common challenge in comparing primary care quality. To address this, attention should be paid to establishing MIDs using a step-by-step framework [30]. Additionally, given that data collection occurred four years ago, further exploration is warranted to understand the persistence or modification of observed associations in the dynamic healthcare landscape, particularly considering the impacts of the COVID-19 pandemic.

## Conclusion

This study highlights the link between patient-perceived primary care quality and self-reported hospital utilisation. Improved first-contact care, comprehensiveness, coordination and patient-centred care are linked to decreased hospital utilisation. Better coordination is associated with a reduced risk of hospital admission, and improved accessibility to primary care is related to lower odds of ED visits. However, the association between continuity of care and increased ED visits warrants further exploration and validation in future research.

## Data Availability

Data are available from the corresponding author upon reasonable request.

## Appendix 1. Detailed sampling approach, inclusion criteria, and informed consent

A multi-phase sampling approach was adopted in this study. In the first phase, purposive sampling was used to select 10 cities, considering economic development, population composition, and healthcare facility models. This included urban and rural areas across the following cities in Guangdong Province, China: Guangzhou, Shenzhen, Zhuhai, Dongguan, Zhongshan, Foshan, Huizhou, Maoming, Meizhou, and Jiangmen. Information regarding the ten cities can be found as below:

**Table:**

City	Per Capita Gross Domestic Product (CNY)	Permanent Population at the end of 2022 (10,000 persons)	Proportion of Urban Population to Permanent Population
Guangzhou	153,625	1873.41	86.5%
Shenzhen	183,274	1766.18	99.8%
Zhuhai	163,654	247.72	90.8%
Foshan	132,517	955.23	95.2%
Dongguan	106,803	1043.70	92.2%
Huizhou	89,157	605.02	72.9%
Zhongshan	81,620	443.11	87.0%
Jiangmen	78,146	482.22	67.9%
Maoming	62,685	623.82	45.8%
Meizhou	34,085	385.80	52.7%

Source of data: Guangdong Statistical Yearbook 2023. http://stats.gd.gov.cn/gdtjnj/content/post_4274608.html

In the second phase, purposive sampling was used to select 12 medical institutions encompassing 16 primary care settings from these regions with diverse patterns of primary care practices in the ten cities.

In the third phase, convenience sampling was employed, involving 100 patients interviewed in each institution. Adult patients (aged 18 years or older) who could speak Mandarin or Cantonese, had three or more visits to the current primary care setting, and agreed to participate in this study were considered eligible and enrolled. Patients with mental illness were excluded from this study.

Verbal informed consent was obtained from all patients at the time of recruitment without formal documentation for several reasons. First, the study posed minimal risk to participants, involving a non-invasive data collection process focused on self-reported information. Second, the study was conducted within a primary care setting, allowing for face-to-face interactions with participants, facilitating the acquisition of verbal consent. Additionally, the use of verbal consent mitigated potential barriers to participation, such as literacy challenges or the necessity of signing written documents. Moreover, the constraints of limited time for recruitment further supported the choice of verbal consent as it expedited the consent process while ensuring ethical standards were upheld.

## Appendix 2. Detailed items in the Assessment Survey of Primary Care (ASPC) scale

### First contact

Item 1.1: When you felt unwell (e.g. got a cold, cough, fever, etc.), did you go to see the general practitioner in the first instance?

Item 1.2: When you experienced flare-ups of your chronic diseases, did you go to see the general practitioner in the first instance?

Item 1.3: When you needed health counselling, did you go to see the general practitioner in the first instance?

Item 1.4: When you needed a health check-up or health examination, did you go to see the general practitioner in the first instance?

Item 1.5: When you needed preventive care, did you go to see the general practitioner in the first instance?.

### Accessibility

Item 2.1: If necessary, could you see the general practitioner during office hours?

Item 2.2: How long did you wait outside the consultation room before you could see the general practitioner?

Item 2.3: Did you feel there it took a long time to wait outside the consultation room?

Item 2.4: If necessary, could you see the general practitioner at night?

Item 2.5: If necessary, could you see the general practitioner on weekends?

Item 2.6: If necessary, could you see the general practitioner at home?

### Continuity

Item 3.1: How many years have you been seeing the general practitioner?

Item 3.2: Did you often see the same general practitioner when you went to the primary care setting for counselling?

Item 3.3: Did you often see the same general practitioner when you went to the primary care setting for prescription?

Item 3.4: Did the general practitioner have a comprehensively knowledge about your medical history?

Item 3.5: Did the general practitioner know your family members?

Item 3.6: Did the general practitioner know about your family members’ medical history?

Item 3.7: Did the general practitioner take the initiative to schedule a follow-up with you?

### Comprehensiveness

Item 4.1: Did you often receive tailored nutrition advice from the general practitioner?

Item 4.2: Did you often receive tailored exercise advice from the general practitioner?

Item 4.3: Did you often receive tailored smoking advice from the general practitioner?

Item 4.4: Did you often receive tailored psychosocial support from the general practitioner?

Item 4.5: Did you often receive tailored health screening advice from the general practitioner?

Item 4.6: Did you often receive tailored cancer screening advice from the general practitioner?

Item 4.7: Did you often receive tailored vaccination advice from the general practitioner?

Item 4.8: Did you often receive community-based health advice from the general practitioner?

### Coordination

Item 5.1: Did you often consult your general practitioner if you needed to transfer to the hospital?

Item 5.2: Did you often inform your general practitioner about your previous treatment plan at the hospital?

Item 5.3: Did your general practitioner often take the initiative to ask about your previous treatment plan at the hospital?

Item 5.4: Did your general practitioner often discuss with you the reason for your transferral?

Item 5.5: Did your general practitioner often discuss which hospital to transfer to with you?

Item 5.6: Did your general practitioner often discuss which department to transfer to with you?

Item 5.7: Did your general practitioner often contact the hospital for you to transfer?

Item 5.8: Did your general practitioner often provide your complete medical record in the transferral letter?

### Patient-centred care

Item 6.1: Did the general practitioner care for you?

Item 6.2: Was the information from the general practitioner enough for you?

Item 6.3: Did you feel understood by the general practitioner?

Item 6.4: Did the general practitioner involve you in decisions about investigations and treatment?

Item 6.5: Was the advice provided by the general practitioner helpful?

Item 6.6: How was your relationship with the general practitioner?

Item 6.7: Could services provided by the general practitioner and the community health centres satisfy most of your health needs?

## Appendix 3. Patient characteristics among the ten cities in Guangdong Province

**Table:**

Patient characteristics		Guangzhou (*N* = 185)		Shenzhen (*N* = 172)		Zhuhai (*N* = 96)		Foshan (*N* = 90)		Dongguan (*N* = 113)		Huizhou (*N* = 126)		Zhongshan (*N* = 102)		Jiangmen (*N* = 111)		Meizhou (*N* = 93)		Maoming (*N* = 97)		*p*
		N	%	N	%	N	%	N	%	N	%	N	%	N	%	N	%	N	%	N	%	
Gender	Male	69	37.3	71	41.3	41	42.7	27	30.0	45	39.8	51	40.5	26	25.5	50	45.0	36	38.7	35	36.1	0.125
	Female	116	62.7	101	58.7	55	57.3	63	70.0	68	60.2	75	59.5	76	74.5	61	55.0	57	61.3	62	63.9	
Age (years)	18–30	4	2.2	42	24.4	14	14.6	11	12.2	17	15	40	31.7	11	10.8	29	26.1	9	9.7	10	10.3	<0.001
	31–50	15	8.1	76	44.2	48	50	36	40.0	63	55.8	57	45.2	36	35.3	40	36.0	21	22.6	17	17.5	
	51–70	102	55.1	41	23.8	29	30.2	30	33.3	21	18.6	27	21.4	39	38.2	25	22.5	42	45.2	36	37.1	
	>70	64	34.6	13	7.6	5	5.2	13	14.4	12	10.6	2	1.6	16	15.7	17	15.3	21	22.6	34	35.1	
Marital status	Unmarried	4	2.2	26	15.1	3	3.1	0	0.0	6	5.3	15	11.9	2	2	18	16.2	1	1.1	6	6.2	<0.001
	Married, divorced or widowed	181	97.8	146	84.9	93	96.9	90	100.0	107	94.7	111	88.1	100	98	93	83.8	92	98.9	91	93.8	
Education	Primary school or below	37	20	14	8.1	19	19.8	27	30.0	23	20.4	23	18.3	32	31.4	41	36.9	40	43	58	59.8	<0.001
	Middle /high school	121	65.4	70	40.7	55	57.3	49	54.4	77	68.1	81	64.3	50	49	55	49.5	51	54.8	36	37.1	
	Bachelor’s degree or above	27	14.6	88	51.2	22	22.9	14	15.6	13	11.5	22	17.5	20	19.6	15	13.5	2	2.2	3	3.1	
Employment status	Employed	22	11.9	108	62.8	58	60.4	38	42.2	83	73.5	91	72.2	37	36.3	68	61.3	35	37.6	38	39.2	<0.001
	Retired	147	79.5	42	24.4	16	16.7	17	18.9	5	4.4	12	9.5	27	26.5	14	12.6	7	7.5	8	8.2	
	Unemployed	16	8.6	22	12.8	22	22.9	35	38.9	25	22.1	23	18.3	38	37.3	29	26.1	51	54.8	51	52.6	
Monthly household Income (CNY)	≤5,000	152	82.2	79	45.9	71	74	61	67.8	85	75.2	94	74.6	75	73.5	91	82.0	87	93.5	93	95.9	<0.001
	5001–10,000	28	15.1	45	26.2	14	14.6	13	14.4	18	15.9	19	15.1	14	13.7	15	13.5	4	4.3	4	4.1	
	>10,000	5	2.7	48	27.9	11	11.5	16	17.8	10	8.8	13	10.3	13	12.7	5	4.5	2	2.2	0	0	
Household status	Local residence	153	82.7	65	37.8	47	49	55	61.1	35	31	21	16.7	57	55.9	63	56.8	91	97.8	95	97.9	<0.001
	Non-local residence	32	17.3	107	62.2	49	51	35	38.9	78	69	105	83.3	45	44.1	48	43.2	2	2.2	2	2.1	
Health insurance	Yes	179	96.8	150	87.2	91	94.8	73	81.1	101	89.4	105	83.3	84	82.4	89	80.2	89	95.7	94	96.9	<0.001
	No	6	3.2	22	12.8	5	5.2	17	18.9	12	10.6	21	16.7	18	17.6	22	19.8	4	4.3	3	3.1	
Health status	Good	52	28.1	79	45.9	37	38.5	43	47.8	53	46.9	67	53.2	46	45.1	56	50.5	25	26.9	31	32	<0.001
	General	110	59.5	88	51.2	45	46.9	39	43.3	42	37.2	45	35.7	48	47.1	42	37.8	46	49.5	34	35.1	
	Poor	23	12.4	5	2.9	14	14.6	8	8.9	18	15.9	14	11.1	8	7.8	13	11.7	22	23.7	32	33	
Number of chronic diseases	0	23	12.4	110	64	60	62.5	62	68.9	69	61.1	87	69	53	52	80	72.1	52	55.9	53	54.6	<0.001
	1	69	37.3	47	27.3	23	24	21	23.3	37	32.7	32	25.4	39	38.2	28	25.2	24	25.8	29	29.9	
	2 and above	93	50.3	15	8.7	13	13.5	7	7.8	7	6.2	7	5.6	10	9.8	3	2.7	17	18.3	15	15.5	
Number of years since first visit to the primary care setting	< 1	5	2.7	40	23.3	9	9.4	15	16.7	8	7.1	9	7.1	12	11.8	13	11.7	6	6.5	2	2.1	<0.001
	1–5	53	28.6	96	55.8	38	39.6	33	36.7	40	35.4	68	54	24	23.5	30	27.0	29	31.2	29	29.9	
	> 5	127	68.6	36	20.9	49	51	42	46.7	65	57.5	49	38.9	66	64.7	68	61.3	58	62.4	66	68	
Number of visits to the primary care setting in the past two weeks	0	23	12.4	19	11	17	17.7	7	7.8	7	6.2	28	22.2	12	11.8	16	14.4	21	22.6	23	23.7	<0.001
	1	101	54.6	131	76.2	54	56.3	56	62.2	89	78.8	80	63.5	69	67.6	67	60.4	41	44.1	51	52.6	
	2 and above	61	33	22	12.8	25	26	27	30.0	17	15	18	14.3	21	20.6	28	25.2	31	33.3	23	23.7	
Whether have a family doctor	No	53	28.6	146	84.9	45	46.9	52	57.8	77	68.1	69	54.8	79	77.5	103	92.8	56	60.2	67	69.1	<0.001
	Yes	132	71.4	26	15.1	51	53.1	38	42.2	36	31.9	57	45.2	23	22.5	8	7.2	37	39.8	30	30.9	

*N = numbers; % = percentages; #*p* values associated with Pearson’s chi-square tests.

## Appendix 4. Patient self-reported hospital utilisation among the ten cities in Guangdong Province

**Table:**

Self-reported hospital utilisation types		Guangzhou		Shenzhen		Zhuhai		Foshan		Dongguan		Huizhou		Zhongshan		Jiangmen		Meizhou		Maoming		*p* value
		(*N* = 185)		(*N* = 172)		(*N* = 96)		(*N* = 90)		(*N* = 113)		(*N* = 126)		(*N* = 102)		(*N* = 111)		(*N* = 93)		(*N* = 97)		
		N	%	N	%	N	%	N	%	N	%	N	%	N	%	N	%	N	%	N	%	
Overall hospital utilisation	No	112	60.5	110	64.0	69	71.9	57	63.3	79	69.9	88	69.8	59	57.8	80	72.1	62	66.7	71	73.2	0.152
	Yes	73	39.5	62	36.0	27	28.1	33	36.7	34	30.1	38	30.2	43	42.2	31	27.9	31	33.3	26	26.8	
Outpatient visits	No	140	75.7	118	68.6	78	81.3	62	68.9	85	75.2	98	77.8	66	64.7	88	79.3	74	79.6	86	88.7	0.002
	Yes	45	24.3	54	31.4	18	18.8	28	31.1	28	24.8	28	22.2	36	35.3	23	20.7	19	20.4	11	11.3	
Hospital admissions	No	172	93.0	167	97.1	87	90.6	85	94.4	110	97.3	119	94.4	98	96.1	102	91.9	86	92.5	81	83.5	0.002
	Yes	13	7.0	5	2.9	9	9.4	5	5.6	3	2.7	7	5.6	4	3.9	9	8.1	7	7.5	16	16.5	
Emergency department visits	No	177	95.7	168	97.7	95	99.0	90	100.0	111	98.2	126	100.0	101	99.0	108	97.3	93	100.0	97	100.0	0.046
	Yes	8	4.3	4	2.3	1	1.0	0	0.0	2	1.8	0	0.0	1	1.0	3	2.7	0	0.0	0	0.0	

*N = numbers; % = percentages; #*p* values associated with Pearson’s chi-square tests.

## Appendix 5. Patient-perceived quality of primary care using the Assessment Survey of Primary Care scale among the ten cities in Guangdong Province

**Table:**

ASPC scale	Guangzhou	Shenzhen	Zhuhai	Foshan	Dongguan	Huizhou	Zhongshan	Jiangmen	Meizhou	Maoming
First-contact care	3.35 ± 0.63	2.73 ± 0.68	3.12 ± 0.65	3.00 ± 0.52	3.08 ± 0.56	3.09 ± 0.55	3.08 ± 0.65	2.78 ± 0.63	3.22 ± 0.55	3.17 ± 0.64
Continuity	2.70 ± 0.67	2.20 ± 0.64	2.28 ± 0.68	2.46 ± 0.62	2.43 ± 0.68	2.21 ± 0.63	2.75 ± 0.66	2.29 ± 0.72	2.85 ± 0.61	2.50 ± 0.66
Accessibility	2.91 ± 0.69	3.02 ± 0.74	3.29 ± 0.61	2.71 ± 0.75	3.03 ± 0.56	3.59 ± 0.43	3.31 ± 0.50	3.38 ± 0.61	3.36 ± 0.54	3.33 ± 0.58
Comprehensiveness	2.38 ± 0.65	2.36 ± 0.64	2.37 ± 0.67	2.21 ± 0.62	2.42 ± 0.67	2.31 ± 0.78	2.52 ± 0.73	2.36 ± 0.56	2.42 ± 0.59	2.47 ± 0.64
Coordination	2.74 ± 0.63	2.54 ± 0.53	2.73 ± 0.44	2.52 ± 0.45	2.73 ± 0.48	2.75 ± 0.53	2.76 ± 0.56	2.70 ± 0.41	2.89 ± 0.47	2.77 ± 0.48
Patient-centred care	3.26 ± 0.57	2.93 ± 0.65	2.90 ± 0.69	2.98 ± 0.60	2.86 ± 0.75	2.80 ± 0.63	3.15 ± 0.64	2.96 ± 0.62	3.06 ± 0.54	2.92 ± 0.61
Total score	2.89 ± 0.45	2.63 ± 0.43	2.78 ± 0.41	2.65 ± 0.40	2.76 ± 0.47	2.79 ± 0.43	2.93 ± 0.49	2.74 ± 0.40	2.97 ± 0.36	2.86 ± 0.39

Note: ^Values are means ± standard deviations.
